# The TRPA1 Channel in the Cardiovascular System: Promising Features and Challenges

**DOI:** 10.3389/fphar.2019.01253

**Published:** 2019-10-18

**Authors:** Zhen Wang, Di Ye, Jing Ye, Menglong Wang, Jianfang Liu, Huimin Jiang, Yao Xu, Jishou Zhang, Jiangbin Chen, Jun Wan

**Affiliations:** ^1^Department of Cardiology, Renmin Hospital of Wuhan University, Wuhan, China; ^2^Cardiovascular Research Institute, Wuhan University, Wuhan, China; ^3^Hubei Key Laboratory of Cardiology, Wuhan, China

**Keywords:** TRPA1 channel, atherosclerosis, heart failure, myocardial ischemia–reperfusion injury, myocardial fibrosis, arrhythmia, vasodilation, hypertension

## Abstract

The transient receptor potential ankyrin 1 (TRPA1) channel is a calcium-permeable nonselective cation channel in the plasma membrane that belongs to the transient receptor potential (TRP) channel superfamily. Recent studies have suggested that the TRPA1 channel plays an essential role in the development and progression of several cardiovascular conditions, such as atherosclerosis, heart failure, myocardial ischemia–reperfusion injury, myocardial fibrosis, arrhythmia, vasodilation, and hypertension. Activation of the TRPA1 channel has a protective effect against the development of atherosclerosis. Furthermore, TRPA1 channel activation elicits peripheral vasodilation and induces a biphasic blood pressure response. However, loss of channel expression or blockade of its activation suppressed heart failure, myocardial ischemia–reperfusion injury, myocardial fibrosis, and arrhythmia. In this paper, we review recent research progress on the TRPA1 channel and discuss its potential role in the cardiovascular system.

## Introduction

The mammalian transient receptor potential (TRP) superfamily of cation channels has six subfamilies, including the vanilloid (TRPV), canonical (TRPC), melastatin (TRPM), ankyrin (TRPA), polycystin (TRPP), and mucolipin (TRPML) subfamilies, which are separated based on sequence homology (Venkatachalam and Montell, 2007). Also known as ATKTM1, the TRPA1 channel represents the sole member of the TRPA subfamily and contains six transmembrane domain (S1–S6) polypeptide subunits. Its name is derived from the unusually high number of ankyrin repeats within the N-terminus and the pore-forming selectivity filter positions between the S5 and S6 transmembrane segments (Story et al., 2003; Nilius and Owsianik, 2011). Since it is a nonselective cation channel, Ca^2+^ and Na^+^ can cross the TRPA1 channel, which results in membrane depolarization and action potential discharge (Zygmunt and Hogestatt, 2014). The TRPA1 channel has been demonstrated to play a pivotal role in mediating a series of pathophysiological reactions, including pain, inflammation, itching, and tissue injury and repair (Bautista et al., 2006; Nilius and Szallasi, 2014; Wang et al., 2018a).

Initially, the TRPA1 channel was thought to be predominately expressed in the sensory neurons of the nodose ganglia, dorsal root ganglia, and trigeminal ganglia (Story et al., 2003; Nilius et al., 2012), as well as in hair cells (Corey et al., 2004; Nagata et al., 2005). In recent years, various researchers have demonstrated that the TRPA1 channel is widely expressed in nonneuronal cells, such as lung fibroblasts (Mukhopadhyay et al., 2011; Nassini et al., 2012), alveolar epithelial cells (Nassini et al., 2012), cardiomyocytes (Andrei et al., 2016), cardiac fibroblasts (Oguri et al., 2014), arterial endothelial cells (ECs) (Qian et al., 2013), pancreatic beta cells (Cao et al., 2012), enterochromaffin cells (Nozawa et al., 2009), odontoblasts (Egbuniwe et al., 2014), and T cells (Bertin et al., 2016).

The TRPA1 channel responds to a wide range of agonists, including pungent natural compounds, such as allyl isothiocyanate (AITC), cinnamaldehyde, and allicin (Bandell et al., 2004; Jordt et al., 2004; Macpherson et al., 2005); ambient toxins, such as acrolein and nicotine (Andre et al., 2008; Talavera et al., 2009); anesthetic agents, such as propofol and lidocaine (Leffler et al., 2011; Woll et al., 2017); chemical compounds, such as ASP-7663 and optovin (Kokel et al., 2013; Kojima et al., 2014); and a range of endogenous agonists, such as oxidized lipids, nitric oxide (NO), and hydrogen sulfide (H_2_S) (Andersson et al., 2008; Eberhardt et al., 2014). Both intracellular and extracellular Ca^2+^ can directly activate the channel, whose activation can be further strengthened by agonists (Zurborg et al., 2007; Wang et al., 2008). However, the effect of temperature on the activation of the TRPA1 channel is controversial. Some researchers have demonstrated that the TRPA1 channel could be activated by noxious cold stimuli (temperature of <17°C) (Story et al., 2003; Del et al., 2010), whereas other studies showed that cold does not activate the TRPA1 channel or that the cold-induced increase in intracellular Ca^2+^ indirectly activates the TRPA1 channel (Zurborg et al., 2007). Recent studies have indicated that the TRPA1 channel is activated by several substances that are produced during oxidative stress, such as hydrogen peroxide (H_2_O_2_), 4-hydroxynonenal (4-HNE), 4-oxononenal (4-ONE), 4-hydroxyhexenal (4-HHE), and 15-deoxy-delta(12,14)-prostaglandin J(2) ([15d-PGJ(2)]) (Trevisani et al., 2007; Andersson et al., 2008; Taylor-Clark et al., 2008). In addition, frequently used TRPA1 agonists are listed in **Table 1**.

**Table 1:** TRPA1 agonists.

Agonists	Source	EC50	Citations
Allyl isothiocyanate	Mustard	33 μM (mice)	Bandell et al., 2004
(AITC)		11 ± 1 μM (rat)	Macpherson et al., 2005
Cinnamaldehyde (CA)	Cinnamon	100 μM (mice)	Bandell et al., 2004
Allicin	Garlic	1.9 μM (human)	Jordt et al., 2004
		1.3 μM (mice)	
Nicotine	Tobacco	10 μM (mice)	Talavera et al., 2009
Propofol	Anesthetic agents	17 μM (mice)	Woll et al., 2017
Lidocaine	Anesthetic agents	24.0 ± 0.6 mM (human) 5.7 ± 0.2 μM (rat)	Leffler et al., 2011
ASP-7663	Synthetic	0.51 μM (human)	Kokel et al., 2013
		0.50 μM (mice)	
		0.54 μM (rat)	
Optovin	Synthetic.	2 μM (mice)	Kojima et al., 2014
Hydrogen peroxide (H_2_O_2_)	Oxidative stress	230 μM (mice)	Andersson et al., 2008
4-Hydroxynonenal (4-HNE)	Oxidative stress	19.9 μM (mice)	Andersson et al., 2008
4-Oxononenal (4-ONE)	Oxidative stress	1.9 μM (mice)	Andersson et al., 2008
4-Hydroxyhexenal (4-HHE)	Oxidative stress	38.9 μM (mice)	Andersson et al., 2008
15-Deoxy-delta(12,14)-prostaglandin J(2) ([15d-PGJ(2)])	Oxidative stress	5.6 μM (mice)	Andersson et al., 2008

Many chemicals, such as ruthenium red, amiloride, camphor, and menthol, have been shown to block this channel, but they lack specificity. Moreover, small-molecule compounds, such as HC-030031 (McNamara et al., 2007) and AP-18 (Petrus et al., 2007; Defalco et al., 2010), are primary inhibitors that specifically bind to the channel and have been widely used to study TRPA1 channel-mediated pharmacology *in vitro* and *in vivo*. Recently, some researchers have reported additional TRPA1 antagonists with highly selectivity and pharmaceutical properties, including A-967079 (Chen et al., 2011), TCS-5861528 (Wei et al., 2009), compound 10 (Copeland et al., 2014), and compound 31 (Rooney et al., 2014). These new-generation TRPA1 channel antagonists will be valuable for exploring the function and therapeutic utility of TRPA1. Frequently used TRPA1 inhibitors are listed in **Table 2**.

**Table 2:** TRPA1 antagonists.

Agonists	Structures	IC50	Citations
HC-030031	Xanthine derivative	6.2 μM (human) 7.6 μM (rat)	McNamara et al., 2007
AP-18	Oxime	3.1 μM (human) 8.8 μM (rat) 4.5 μM (mice)	Petrus et al., 2007 Defalco et al., 2010
A-967079	Oxime	0.067 μM (human) 0.289 μM (rat)	Chen et al., 2011
TCS-5861528	Xanthine derivative	14.3 μM (human)	Wei et al., 2009
Compound 10	Xanthine derivative	0.17 μM (human) 0.056 μM (rat)	Copeland et al., 2014
Compound 31	Imidazopyridine derivative	0.015 μM (human) 0.089 μM (rat)	Rooney et al., 2014

Previous studies have demonstrated that the TRPA1 channel is also widely expressed in the cardiovascular system and is involved in regulating intracellular Ca^2+^ concentrations (Oguri et al., 2014; Andrei et al., 2016). In this review, we summarize the potential involvement of the TRPA1 channel in modulating pathophysiologic conditions, including atherosclerosis, heart failure, myocardial ischemia–reperfusion injury (IRI), myocardial fibrosis, arrhythmia, vasodilation, and hypertension (**Table 3**).

**Table 3:** Role of the TRPA1 channel in the cardiovascular system.

Diseases	Animals	Cellular localization	Effects	Mechanisms	Citations
Atherosclerosis	Male C57BL/6, apoE^−/−^ and apoE^−/−^TRPA1^−/−^ mice (8 weeks old)	Macrophages	AITC (10 mg/kg/d, 4 weeks, i.g.) suppresses atherosclerosis; HC-030031 (10 mg/kg/d, 4 weeks, i.g.) and TRPA1 knockout exacerbate atherosclerosis	Cholesterol metabolism and inflammation in macrophages	Zhao et al., 2016
Heart failure	Male C57BL/6 mice (8–10 weeks old)	Cardiomyocytes and macrophages	HC-030031 (10 mg/kg/d, 4 weeks, i.g.) and TCS-5861528 (3 mg/kg/d, 4 weeks, i.g.) ameliorate cardiac hypertrophy and heart failure	Inhibits Ca^2+^-dependent signal pathways and macrophage polarization	Wang et al., 2018b
Myocardial IRI	Male Sprague-Dawley rats (8–10 weeks old)	Cardiomyocytes	ASP-7663 (3 mg/kg *in vivo* and 3 μM *in vitro*) and AP-18 (1 mg/kg *in vivo* and 1 mM *in vitro*) reduce myocardial injury, but cinnamaldehyde (0.01 mg/kg *in vivo*) did not affect myocardial infarct size	Pain management and anti-inﬂammatory drugs	Lu et al., 2016
	Male C57BL/6 and TRPA1^−/−^ mice (12–16 weeks old)	Cardiomyocytes	TRPA1 knockout reduces infarct size	Reduces Ca^2+^ overload	Conklin et al., 2019
Myocardial fibrosis	–	Human adult ventricular cardiac fibroblasts	HC-030031 (100 μM) and siRNA targeting the TRPA1 channel inhibit methylglyoxal-induced (300 µM) proliferation of cardiac fibroblasts	Inhibits Ca^2+^ entry	Oguri et al., 2014
Arrhythmia	Female B6129 mice (19–21 weeks old) and TRPA1^−/−^ mice (21–28 weeks old)	–	Acrolein (537 ppm, 8 times/4 weeks, inhalation) increases heart rate variability and myocardial desynchrony in B6129 mice but not in TRPA1^−/−^ mice	Influence the autonomic nervous system	Thompson et al., 2019
	Female C57BL/6 and TRPA1^−/−^ mice (15–30 weeks old)	–	TRPA1 knockout decreases acrolein-induced (3 ppm, 3 h) heart rate variability and arrhythmias	Cardiac autonomic function	Kurhanewicz et al., 2016 Kurhanewicz et al., 2018
	Male spontaneously hypertensive rats (18–20 weeks old)	–	HC-030031 (5 mg/kg, i.p.) reduces diesel exhaust (32 ppm, 4 h)- and aconitine (1.5 mg/kg, i.p.)-induced ventricular arrhythmias	Restrains the activity of sympathetic and autonomic imbalance	Hazari et al., 2011
	Male Sprague-Dawley rats (15 weeks old)	–	AITC (30 mM) inhalation causes bradycardia atrioventricular blockade and prolonged PR intervals	Activates the vagus nerve	Hooper et al., 2016
Vasodilation	Female CD1, C57BL/6, CGRP^−/−^, TRPV1^−/−^, and TRPA1^−/−^ mice	–	4-ONE (1–30 nmol, intraplantar injection) triggers a vasodilation response, but not in TRPA1^−/−^ mice	TRPA1-dependent neurogenic vasodilatation	Graepel et al., 2011
	Male Sprague-Dawley rats	Trigeminal root ganglia neurons	AITC (100 μM, intranasal administration) and acrolein (30 μM, intranasal administration) increase cerebral blood flow, but the effect is blocked by HC-030031 (50 μM, intranasal administration)	Neurogenic vasodilation	Kunkler et al., 2011
	Male CD1, CGRP^−/−^, TRPV1^−/−^, and TRPA1^−/−^ mice (8–12 weeks old)	–	Cinnamaldehyde (1%–30%) increases the blood flow, but not in HC-030031 (100 mg/kg)-treated and TRPA1 knockout mice	Neurogenic vasodilation	Aubdool et al., 2016
	Male Sprague-Dawley rats	Endothelial cells	AITC-induced (3–100 μM) cerebral artery dilation was abolished by the administration of HC-030031 (3 μM)	Endothelium-dependent vasodilation	Earley et al., 2009
	Adolescent rats	Endothelial cells	AITC (15–60 μM) evokes graded cerebral artery vasodilation	Endothelium-dependent vasodilation	Qian et al., 2013
Hypertension	Male C57BL/6 and TRPA1^−/−^ mice (8–12 weeks old)		Conscious C57BL/6 and TRPA1^−/−^ mice have similar basal blood pressures and heart rates	–	Bodkin et al., 2014
	CD1, CGRP^−/−^, TRPV1^−/−^, and TRPA1^−/−^ mice	–	Cinnamaldehyde (80–320 μM/kg) induces a transient hypotensive response followed by a sustained hypertensive response	Autonomic system reflexes	Pozsgai et al., 2010
	Male Sprague-Dawley rats (15 weeks old)	–	AITC (30 mM) induces a transient hypertensive response followed by a prolonged hypotensive response	Autonomic system reflexes	Hooper et al., 2016

## Role of the Trpa1 Channel in the Cardiovascular System

### Atherosclerosis

Atherosclerosis and its complications remain the leading causes of morbidity and mortality in developed countries (Momiyama et al., 2014; Solanki et al., 2018). Atherosclerosis, characterized by the hardening of the arterial wall and the narrowing of the arterial lumen, is considered a chronic inflammatory disease that results from lipid metabolism dysfunction, smooth muscle cell proliferation, and cholesterol-laden macrophage accumulation (Khera et al., 2011; Moore et al., 2013; Ye et al., 2017b). Evidence supports the idea that macrophages are the dominant immune cells and play critical roles in the development of atherosclerosis. During the early stages of atherosclerosis, the accumulation of lipid-laden macrophages and oxidized low-density lipoprotein (oxLDL) leads to lipid droplet generation in the subendothelial space. Macrophage accumulation and foam cell formation exacerbate the development of unstable plaques and plaque rupture (Libby, 2002; Khan et al., 2015).

As we know, cholesterol homeostasis in macrophage foam cells is regulated by the complex mechanisms underlying oxLDL internalization and cholesterol efflux. In macrophage foam cells, oxLDL internalization is mediated by scavenger receptor transporters, and several macrophage transporters, including ATP-binding cassette subfamily A member 1 (ABCA1) and ABCG1, are responsible for cholesterol efflux. In a mouse high-fat diet-induced atherosclerosis model, TRPA1 was localized mainly in macrophages; TRPA1 channel activation with AITC suppressed the progression of atherosclerosis in apolipoprotein E (apoE)^−/−^ mice, while the protective effect was lost in apoE^−/−^TRPA1^−/−^ mice. Moreover, inhibition of TRPA1 activity exacerbated atherosclerosis and abated cholesterol efflux by suppressing oxLDL-induced cholesterol efflux but did not alter oxLDL internalization (Zhao et al., 2016). This implied that TRPA1 activation suppressed excessive lipid accumulation by increasing ABC transporter-mediated cholesterol efflux. Inflammatory conditions are also a key event an implicated in the progression of atherosclerosis. The author also reported that AITC administration prevented TNF-α-induced macrophage inflammation. Thus, activation of the macrophage TRPA1 channel suppressed atherosclerosis by inhibiting cholesterol efflux and the inflammatory response. In all, these data indicate that pharmacological activation of the TRPA1 channel may have therapeutic value to prevent or treat atherosclerosis.

The existing research is very creative and interesting, but many crucial points still need consideration. Macrophage-mediated cholesterol metabolism and proinflammatory cytokine secretion are central steps in the initiation and progression of atherosclerosis. While there is considerable knowledge on the functions of Ca^2+^ influx from the endothelium and macrophages in atherosclerosis, less is known about the molecular mechanisms by which the TRPA1 channel regulates the formation of macrophage-derived foam cells that directly or indirectly depend on Ca^2+^ influx. Moreover, the macrophage number, macrophage phenotype, macrophage apoptosis, and lesion autophagy are also important determinants of atherosclerosis progression. Whether the TRPA1 channel modulates both the recruitment and polarization of macrophages toward a pro- or anti-inflammatory phenotype is unclear. Further studies require the generation of macrophage-specific TRPA1 knockout mice to examine the contribution of the TRPA1 channel to atherogenesis.

### Heart Failure

Heart failure is a complex and multisystem clinical syndrome that results from impaired ventricular systolic and/or diastolic function (Katz and Rolett, 2016). Heart failure is a result of exposure to chronic cardiac stress or injury, including pressure overload, myocardial infarction or ischemia, myocarditis, and inherited diseases (Bacmeister et al., 2019; Ye et al., 2019). Ca^2+^ is a critical second messenger in cardiac function. It participates in not only in the excitation–contraction coupling and relaxation of the heart but also in a key signal transduction pathway responsible for various cardiovascular diseases (Goldhaber and Philipson, 2013). Altered Ca^2+^ homeostasis contributes notably to the pathophysiology of heart failure, and many models of heart failure exhibit deficient Ca^2+^ handling in cardiomyocytes (Bers, 2006; Roe et al., 2015). For example, the Ca^2+^-dependent Ca^2+^/calmodulin-dependent protein kinase II (CaMKII) and calcineurin pathways are centrally involved in mediating pathological hypertrophy and heart failure (Molkentin et al., 1998; Anderson et al., 2011). While the precise mechanism remains unclear, accumulating evidence suggests that targeting Ca^2+^ homeostasis in failing cardiomyocytes presents a promising new therapeutic approach for improving cardiac function.

Our previously study showed that TRPA1 expression is increased in failing human and mouse hearts (Wang et al., 2018b). Inhibition of the TRPA1 channel significantly ameliorated cardiac hypertrophy and heart failure by decreasing the increases in heart weight index and ventricular volume and improving cardiac function in transverse aortic constriction-induced pressure overload in mice. In addition, TRPA1 inhibition significantly reduced critical factors involved in heart failure, including calcineurin and CaMKII phosphorylation in mice induced by pressure overload. Another study demonstrated that that TRPA1 activation induced a dose-dependent increase in contractile function and peak [Ca^2+^]_i_ in cardiomyocytes but not in cardiomyocytes obtained from TRPA1^−/−^ mice (Andrei et al., 2017). In addition, the CaMKII inhibitor KN-93 reversed the AITC-induced increase in [Ca^2+^]_i_ and contractile function. Based on these studies, it may be suggested that TRPA1 participates in the regulation of heart failure *via* a Ca^2+^-dependent mechanism.

**Figure 1:**
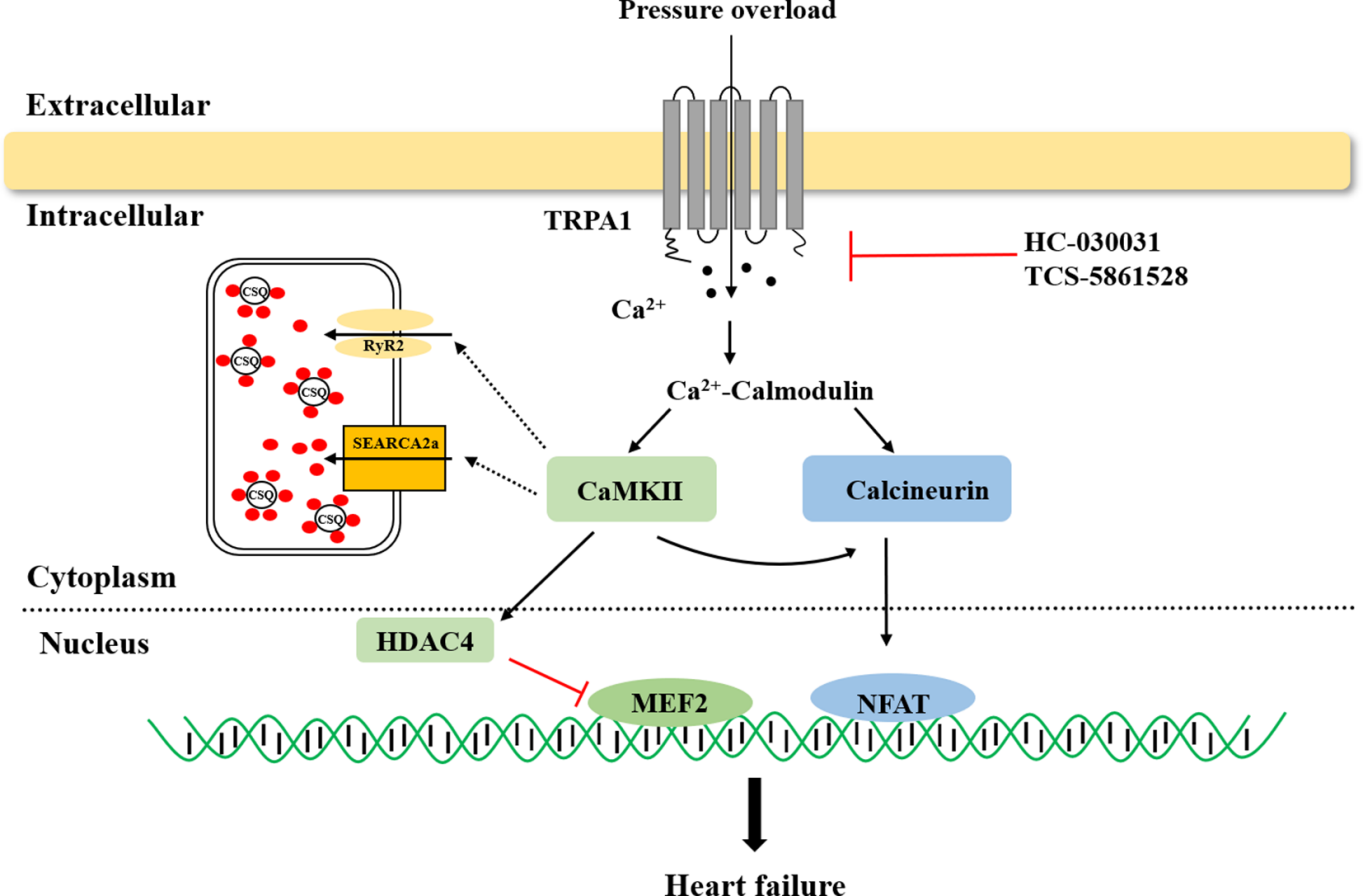
The role of transient receptor potential ankyrin 1 (TRPA1) in heart failure. TRPA1 channel inhibition decreased Ca^2+^ influx and inhibited Ca^2+^-dependent pathway activation, ultimately ameliorating pressure overload induced-heart failure. CaMKII, Ca^2+^/calmodulin-dependent protein kinase II; HDAC4, histone deacetylase 4; MEF2, myocyte enhancer factor 2; NFAT, nuclear factor of activated T cells.

In failing hearts, Ca^2+^ homeostasis is markedly altered, resulting in impaired systolic and diastolic function (Marks, 2013; Roe et al., 2015). Previous research revealed that the TRPA1 channel is present in cardiomyocytes and that TRPA1-mediated Ca^2+^ release contributed to nearly 40% of the overall [Ca^2+^]_i_ increase in the physiological state (Andrei et al., 2016; Shang et al., 2016). The links between TRPA1 and heart failure are briefly summarized in **Figure 1**. Pressure overload activates the TRPA1 channel and increases Ca^2+^ influx. The activation of Ca^2+^-dependent pathways, including CaMKII and calcineurin, leads to hypertrophic gene expression and mediates heart failure.

### Myocardial IRI

Myocardial IRI is a pathological condition that occurs after a critical ischemic period followed by blood supply restoration and reoxygenation that correlates with a deterioration of myocardial function and a marked inflammatory reaction (Ferdinandy et al., 2007; Eltzschig and Eckle, 2011). A variety of pathological processes and mediators, including intracellular Ca^2+^ overload and excess reactive oxygen species (ROS), are proposed to be crucial in myocardial IRI. During ischemia, ion pumps cannot function, resulting in a rise in Ca^2+^, which leads to intracellular Ca^2+^ overload, particularly during reperfusion when oxygen is reintroduced (Murphy and Steenbergen, 2008; Garcia-Dorado et al., 2012). ROS are produced physiologically by the mitochondrial electron transport chain during respiration, and increased ROS can result in mitochondrial matrix swelling and cell death (Murphy and Steenbergen, 2008; Zhou et al., 2015). Studies have shown substantial interest in developing therapies to prevent myocardial IRI. In particular, site-targeted treatments, such as inhibiting Ca^2+^ overload and reducing ROS accumulation, may improve the protective effect on the stressed myocardium (Raedschelders et al., 2012; Jennings, 2013).

The TRPA1 channel is a calcium-permeable nonselective cation channel in the plasma membrane; however, it also functions as a sensor that is activated by ROS and is modulated when intracellular changes in oxygen levels occur, and both factors are important for IRI (Viana, 2016; Pires and Earley, 2017). However, the role and mechanism of TRPA1 in myocardial IRI are conflicting and are still controversial. In an *in vivo* rat model of myocardial IRI (30 min of ischemia followed by 2 h of reperfusion), the administration of the TRPA1 agonist reduced the myocardial infarct size before ischemia and reperfusion, and TRPA1 channel inhibition also blocked the infarct size-sparing effects of morphine. In isolated cardiomyocytes, the activation of the TRPA1 channel reduced cardiomyocyte cell death and the release of lactate dehydrogenase when activated during reoxygenation (Lu et al., 2016). In contrast, global knockout of TRPA1 results in less myocardial injury in a mouse model of IRI (30 min of ischemia followed by 24 h of reperfusion) (Conklin et al., 2019). In addition, acrolein, an IRI-associated toxin, induced Ca^2+^ overload and hypercontraction in isolated cardiomyocytes, which were significantly ameliorated by TRPA1 inhibitor. These studies are contradictory, may be because of the exerting differential effects, TRPA1 in cardiac myocytes, vascular cells, and sensory neurons, which eliminates its function leads to variable effects on IRI. Therefore, a genetically modified mouse model with cell-specific deletion (or targeted inhibition) of TRPA1 in cardiomyocytes will be required to assess the role of myocardial TRPA1 in IRI.

**Figure 2:**
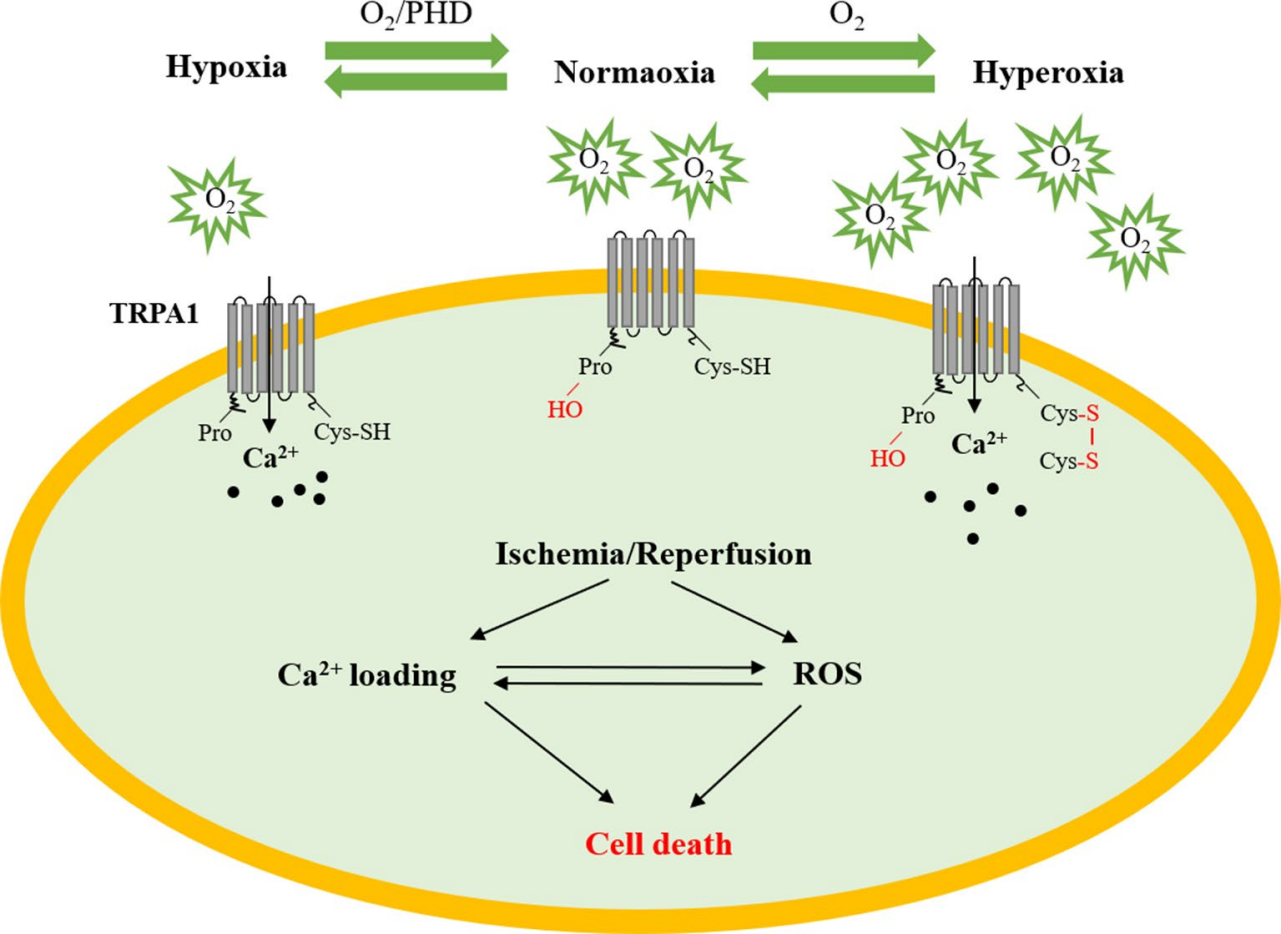
The role of TRPA1 in myocardial ischemia–reperfusion injury. Under normoxic conditions, PHDs inhibit TRPA1 channel activity. Under hyperoxic conditions, O_2_ directly activates the TRPA1 channel through reversible covalent or oxidative modification of cysteine residues. Under hypoxic conditions, the TRPA1 channel was activated by O_2_-dependent inhibition relief by PHD-mediated hydroxylation of a proline residue. The TRPA1 channel participates in the development of myocardial ischemia–reperfusion injury by regulating Ca^2+^ overload and ROS. PHD, prolyl hydroxylases; Cys, cysteine residue; ROS, reactive oxygen species.

As we known, the sensitivity of the TRPA1 channel to ROS was the greatest among the TRP channels (Yamamoto and Shimizu, 2016). Moreover, the TRPA1 channel also plays a pivotal role in the maintenance of O_2_ homeostasis (Takahashi et al., 2011). This study found that O_2_ could activate the TRPA1 channel through reversible covalent or oxidative modification of cysteine residues in hyperoxia directly. In addition, prolyl hydroxylases (PHDs) could inhibit TRPA1 channel activity under the condition of normoxia, whereas hypoxia could activate TRPA1 by diminishing the activity of PHDs. Accumulating research has shown that Ca^2+^ can enhance ROS output and generation, which correlates well with metabolic rate. Thus, it will be very interesting to determine the mechanism by which the TRPA1 channel mediates Ca^2+^ homeostasis and redox signaling in myocardial IRI (**Figure 2**).

### Myocardial Fibrosis

Myocardial fibrosis is associated with cardiac fibroblast overproliferation and excessive extracellular matrix (ECM) protein accumulation in the myocardial interstitium (Schirone et al., 2017; Zhang et al., 2018). Cardiac fibroblasts are activated in response to a variety of pathological stimuli, such as myocardial injury, pressure overload, and failed repair. Cardiac fibroblasts are the main producers of ECM and play an important role in cell signaling and fibrotic responses (Yue et al., 2013; Moore-Morris et al., 2015). Accumulating evidence has demonstrated that Ca^2+^-dependent signaling is essential for the proliferation and differentiation of cardiac fibroblasts and ECM production (He et al., 2011; Yue et al., 2013). However, Ca^2+^-permeable channels in coordination with Ca^2+^-dependent signaling in cardiac fibroblasts are not fully understood. Thus, understanding the molecules responsible for Ca^2+^-dependent signaling in cardiac fibroblasts will provide novel targets for antifibrotic strategies.

Methylglyoxal (MG), a highly reactive dicarbonyl compound, is a metabolic intermediate of glycolysis that can activate TRPA1. MG can confer deleterious cardiovascular effects, which are associated with the activation of fibrosis (Desai and Wu, 2007; Subramanian and Nagarajan, 2017). In an *in vitro* study, TRPA1 was expressed in human adult ventricular cardiac fibroblasts, and inhibiting TRPA1 channel activity reduced MG-induced Ca^2+^ influx. Moreover, inhibition of TRPA1 suppressed MG-induced fibroblast proliferation and increased α-smooth muscle actin expression. In this study, the TRPA1-mediated Ca^2+^-dependent signaling pathway was suggested to be required for MG-induced cell cycle progression and differentiation in human cardiac fibroblasts (Oguri et al., 2014). Another study also demonstrated that inhibition of the TRPA1 channel attenuated fibrosis and inflammation by attenuating TGF-β1 signaling cascades in ocular fibroblasts (Okada et al., 2014). Taken together, these studies indicated that the TRPA1 channel may serve as a potential novel therapeutic target for fibrotic responses.

In recent years, the importance of TRP channels in regulating Ca^2+^ signaling and cardiac fibrogenesis has been recognized (Thodeti et al., 2013). Inhibition of the TRPM7 channel reduced the proliferation and differentiation of cardiac fibroblasts and ECM production (Du et al., 2010). In addition, the TRPC3 channel functions as a critical mediator in myocardial fibrosis in coordination with Ca^2+^ signaling and ROS production (Numaga-Tomita et al., 2017). Compared with most other TRP channels, the TRPA1 channel has high Ca^2+^ permeability (Zygmunt and Hogestatt, 2014). As the sole member of the TRPA subfamily, the TRPA1 channel could be important in causing cardiac fibrosis. Thus, further studies are required to clarify the molecular mechanisms underlying the regulation of myocardial fibrosis by the TRPA1 channel.

### Arrhythmia

Common air pollution is composed of particulate matter and gaseous pollutants, such as ozone, nitrogen oxides, sulfur dioxide, aldehydes, and acrolein. Epidemiological studies have shown that the deleterious effects of noxious irritants and pollutant inhalation occur upon both short-term and long-term exposure. Air pollution can lead to millions of premature deaths worldwide, of which 60%–80% are cardiovascular diseases. In fact, exposure to air pollutants has been well established as a factor that increases the risk for cardiovascular events, particularly increasing the susceptibility to arrhythmias by altering autonomic nervous system (ANS) balance (Brook et al., 2004; Langrish et al., 2014).

Acrolein is a volatile, unsaturated aldehyde and a toxic combustion product present in tobacco smoke and fires (Stevens and Maier, 2008). Recent research reported that exposure to acrolein increased heart rate (HR) variability and the incidence of arrhythmias in WT mice, while these consequences were eliminated in TRPA1^−/−^ mice (Kurhanewicz et al., 2016; Kurhanewicz et al., 2018; Thompson et al., 2019). Moreover, exposure to diesel exhaust (DE) and aconitine increased sympathetic activation in spontaneously hypertensive rats. Pretreatment of low DE-exposed rats with a TRPA1 inhibitor or sympathetic blockade reduced susceptibility to ventricular arrhythmias (Hazari et al., 2011). As we know, HR variability is considered to be a marker of ANS imbalance and a risk factor for cardiovascular events. These findings likely indicated that the TRPA1 channel may contribute to the proarrhythmic response by causing sympathetic activation and ANS imbalance.

Other studies showed that the activation of the TRPA1 channel evoked reflex-mediated increases in parasympathetic activity. The application of AITC increased HR and renal sympathetic nerve activity, which were blunted in rats with chronic heart failure (Adam et al., 2019). Exposure of conscious rats to AITC caused a significant increase in the incidence of arrhythmic events, which included bradycardia, atrioventricular blockade, and even prolonged PR intervals. Furthermore, such responses to AITC exposure were inhibited by the cholinergic antagonist atropine (Hooper et al., 2016).

The ANS is composed of the sympathetic and parasympathetic systems located throughout the body, including in the heart, lung, and vasculature system. These sensory nerves are sensitive to multiple stimuli and responsible for maintaining homeostasis by having either excitatory or inhibitory effects. The responses to air pollutants or irritant inhalation are associated with the activation of the sympathetic and parasympathetic systems, which do not function independently and are highly integrated (Middlekauff et al., 2014; Perez et al., 2015). Previous studies have shown that TRPA1 channels are expressed in primary sensory neurons and that TRPA1 activation results in both afferent and efferent signaling (Zygmunt and Hogestatt, 2014). Different air pollutants and irritants have different effects when TRPA1 is activated, related to the activation of the sympathetic or parasympathetic systems. Thus, considering the complexity of the composition of air pollution and irritants, further studies are needed to identify the roles of the TRPA1 channel in modulating autonomic control of the heart.

### Vasodilation

The vascular system plays essential roles in the transport of gases (such as oxygen and carbon dioxide), nutrients (such as amino acids and electrolytes), and circulating cells in the organism. The vascular system is an exquisitely sculpted vascular network throughout the body and contains multiple components. Among them, ECs and smooth muscle cells act as vital mediators of vascular homeostasis maintenance. In addition, perivascular sensory neurons contribute to the regulation of vascular tone through the release of neuropeptides, such as calcitonin gene-related peptide (CGRP) and substance P (Aubdool et al., 2016).

Recent studies demonstrated the involvement of the TRPA1 channel in the regulation of vasodilation (Earley, 2012; Eberhardt et al., 2014). Intraplantar injection of 4-ONE triggered a significant vasodilation response, which was absent in TRPA1^−/−^ and CGRP^−/−^ mice (Graepel et al., 2011). Moreover, AITC and environmental irritants, such as formaldehyde and acrolein, stimulate CGRP release and increase cerebral blood flow, effects that were blocked by TRPA1 and a CGRP receptor antagonist (Kunkler et al., 2011). Topical application of cinnamaldehyde to mouse ears resulted in a marked increase in the blood flow in the skin but not in HC-030031- or nitric oxide synthase (NOS) antagonist-pretreated mice (Aubdool et al., 2016). Based on the findings of these studies, it may be proposed that the activation of the TRPA1 channel could mediate neurogenic vasodilation in the peripheral vasculature and could be mediated by the sensory neuropeptide CGRP and NOS-derived NO.

Other studies also demonstrated that the TRPA1 channel causes vasodilation through an endothelium-dependent mechanism involving Ca^2+^ influx in arterial ECs. Earley and colleagues showed that TRPA1 channel was present in arterial ECs, and the AITC-induced cerebral artery dilation was abolished by TRPA1 inhibition and endothelium disruption. In addition, TRPA1 activation-induced dilation was suppressed by treatment with the small and intermediate conductance Ca^2+^-activated K^+^ channel (K_Ca_) and an inwardly rectifying K^+^ channel (K_IR_) blocker (Earley et al., 2009). Moreover, activation of the TRPA1 channel enhanced endothelial Ca^2+^ dynamics primarily through the recruitment of endothelial Ca^2+^ and evoked graded cerebral artery vasodilation (Qian et al., 2013).

**Figure 3:**
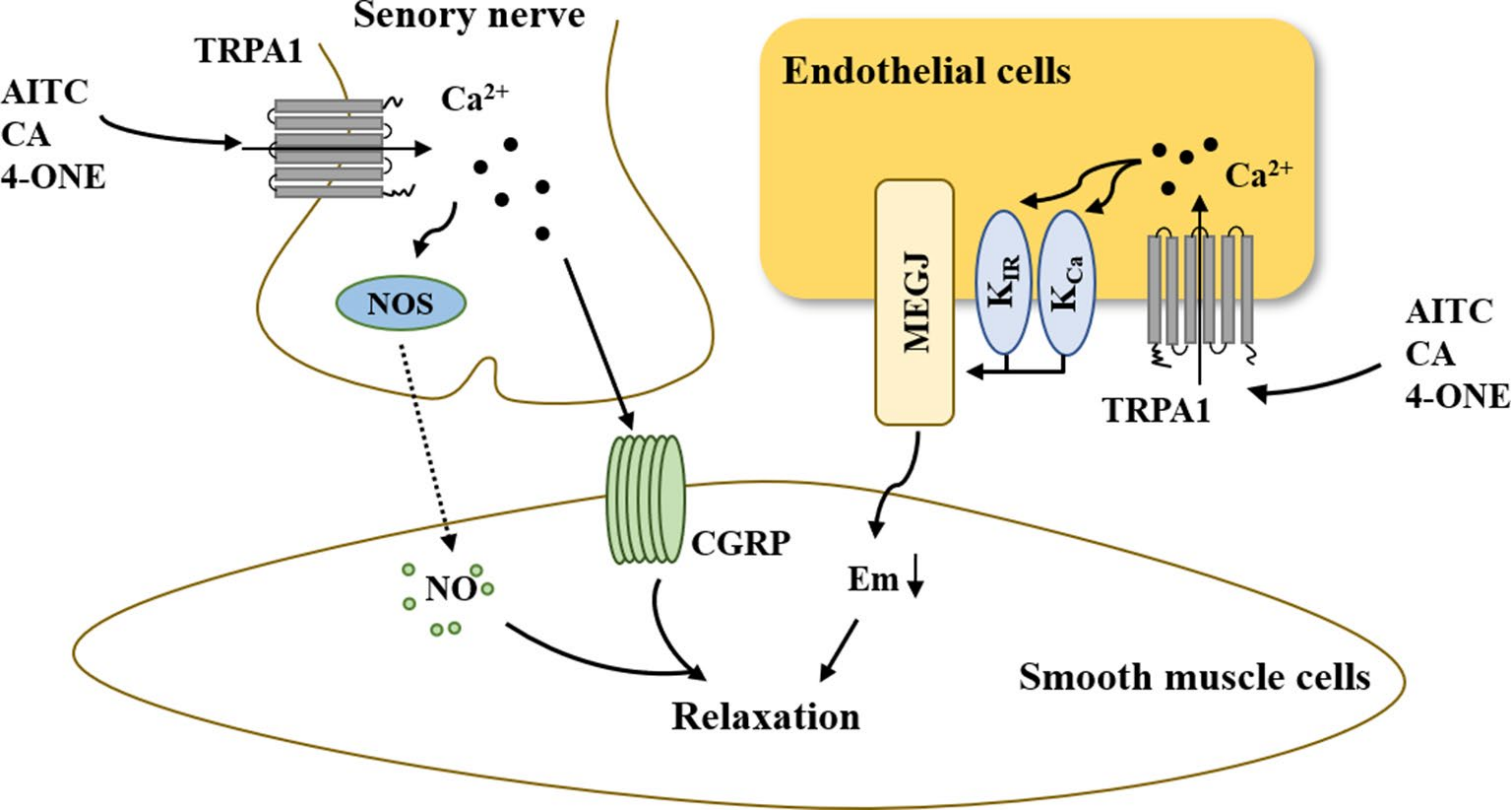
The role of TRPA1 in vasodilation. On the one hand, increased Ca^2+^ influx *via* TRPA1 activation results in hyperpolarization of the endothelial cell plasma membrane. The change in membrane potential is occurs to hyperpolarize the vascular smooth muscle cell plasma membrane, resulting in myocyte relaxation. On the other hand, the activation of TRPA1 in sensory neurons induces an increase in [Ca^2+^]_i_, leading to the release of the neuropeptide CGRP and NOS-derived NO, thus mediating vasodilation. AITC, allyl isothiocyanate; CA, cinnamaldehyde; 4-ONE, 4-oxo-2-nonenal; CGRP, calcitonin gene-related peptide; NOS, nitric oxide synthase; NO, nitric oxide; K_Ca_, Ca^2+^-activated K^+^ channel; K_IR_, inwardly rectifying K^+^ channel; MEGJs, myoendothelial gap junctions.

In summary, the above-mentioned results demonstrated that activation of the TRPA1 channel can elicit peripheral vasodilation and that the main mechanisms underlying this process involve nerve-induced vasodilation and endothelium-dependent vasodilation (**Figure 3**). On the one hand, the increased Ca^2+^ influx *via* TRPA1 activation resulted in hyperpolarization of the endothelial cell plasma membrane. The change in membrane potential occurs to hyperpolarize the vascular smooth muscle cell plasma membrane, resulting in myocyte relaxation. On the other hand, activation of TRPA1 in sensory neurons induces an increase in [Ca^2+^]_i_, leading to the release of the neuropeptide CGRP and NOS-derived NO, thus mediating vasodilation.

### Hypertension

Hypertension, characterized by increases in systolic blood pressure and/or diastolic blood pressure, is a common clinical disorder and a major public health issue (Oparil and Schmieder, 2015; Ye et al., 2017a; Taler, 2018). Hypertension is also a major risk factor for coronary artery disease, heart failure, stroke, and chronic kidney disease (Lau et al., 2017; Taler, 2018). Despite significant advances in antihypertensive therapy, a substantial proportion of patients have uncontrolled blood pressure. As such, there is a continued need to identify new targets to control blood pressure effectively.

Examination of whole-body TRPA1-deficient mice demonstrated that there was no difference in the baseline mean arterial pressure or HR of WT and TRPA1^−/−^ mice under anesthesia (Pozsgai et al., 2010). Conscious C57BL/6 and TRPA1^−/−^ mice have similar morphologic and hemodynamic parameters, including basal blood pressure and HR. In addition, similar blood pressure and HR were also observed after angiotensin II infusion (Bodkin et al., 2014). These studies suggest that mice can maintain basic blood pressure and compensate for TRPA1 deficiency.

Experimental and clinical investigations have tested the key role played by the ANS in modulating cardiovascular functions and controlling blood pressure. In humans and experimental animals, both the increased sympathetic nerve tension and reduced parasympathetic tone activity are associated with and responsible for the appearance and maintenance of hypertension and hypertension-related sequelae (Folkow, 1982; Mancia and Grassi, 2014). Intravenously injected TRPA1 agonist cinnamaldehyde induced a transient hypotensive response followed by a sustained hypertensive response in anesthetized mice. A lower dose of cinnamaldehyde (80 μM/kg) induced a hypotensive response that was significantly less than that in TRPA1^−/−^ mice. Interestingly, the hypertensive response associated with a higher cinnamaldehyde dose (320 μM/kg) was blunted in TRPA1^−/−^ mice. In addition, the cholinergic antagonist atropine significantly inhibited the hypotensive response to the low dose of cinnamaldehyde. The α-adrenergic blocker prazosin significantly inhibited both the hypotensive and hypertensive responses (Pozsgai et al., 2010). Another study showed that inhalation of AITC induced a transient hypertensive response followed by a prolonged hypotensive response in SD rats. Intraperitoneal injection of atropine accentuated the AITC-induced hypertensive response and prevented a hypotensive response. However, pretreatment with terazosin, an α1-adrenergic blocker, prevented the AITC-induced hypertensive response (Hooper et al., 2016). These data indicate that the TRPA1 channel can also influence changes in blood pressure *via* reflex modulation of the ANS.

Therefore, although mice are able to compensate for TRPA1 deficiency and maintain basic blood pressure, the TRPA1 channel can also influence changes in blood pressure *via* reflex modulation of the ANS. Given the diverse activation mechanisms of the TRPA1 channel, future studies may uncover more important functions of the channel in blood pressure regulation. Thus, defining the role of the TRPA1 channel in the regulation of blood pressure will provide a new target for future investigations of antihypertensive therapies.

## Conclusion

The current review described the potential role of the TRPA1 channel in the regulation of the cardiovascular system. The TRPA1 channel is expressed in the cardiovascular system and involved in mediating a series of cardiovascular pathophysiologies. Activation of the TRPA1 channel has a protective effect against the development of atherosclerosis. Furthermore, TRPA1 channel activation elicits peripheral vasodilation and induces a biphasic blood pressure response. However, loss of channel expression or blockade of its activation suppressed heart failure, myocardial IRI, myocardial fibrosis, and arrhythmia. This finding indicates that the TRPA1 channel can modulate cardiovascular diseases in both positive and negative manners. Given the link between the TRPA1 channel and various cardiovascular diseases, it could be an attractive drug target for therapeutic interventions. However, we should also pay attention to the dual functions of the TRPA1 channel when designing clinical experiments. For this reason, more basic research on the function of the TRPA1 channel must be conducted before TRPA1 channel modulators are used in the clinical setting.

## Author Contributions

ZW conceived the review and drafted the manuscript. ZW, DY, JY, MW, JL, HJ, YX, JZ, JC, and JW revised the manuscript critically for important intellectual content. All authors approved the final version of the manuscript submitted. In addition, ZW wants to thank, in particular, the patience, care, and support from Lin Tian over the past years: “Grow old along with me, the best is yet to be.”

## Conflict of Interest

The authors declare that the research was conducted in the absence of any commercial or financial relationships that could be construed as a potential conflict of interest.

## References

[B1] Adam R. J. Xia Z. Pravoverov K. Hong J. Case A. J. Schultz H. D. (2019). Sympathoexcitation in response to cardiac and pulmonary afferent stimulation of TRPA1 channels is attenuated in rats with chronic heart failure. Am. J. Physiol. Heart Circ. Physiol. 316, H862–H872. 10.1152/ajpheart.00696.2018 30707612 PMC6483016

[B2] Anderson M. E. Brown J. H. Bers D. M. (2011). CaMKII in myocardial hypertrophy and heart failure. J. Mol. Cell. Cardiol. 51, 468–473. 10.1016/j.yjmcc.2011.01.012 21276796 PMC3158288

[B3] Andersson D. A. Gentry C. Moss S. Bevan S. (2008). Transient receptor potential A1 is a sensory receptor for multiple products of oxidative stress. J. Neurosci. 28, 2485–2494. 10.1523/JNEUROSCI.5369-07.2008 18322093 PMC2709206

[B4] Andre E. Campi B. Materazzi S. Trevisani M. Amadesi S. Massi D. (2008). Cigarette smoke-induced neurogenic inflammation is mediated by alpha,beta-unsaturated aldehydes and the TRPA1 receptor in rodents. J. Clin. Invest. 118, 2574–2582. 10.1172/JCI34886 18568077 PMC2430498

[B5] Andrei S. R. Ghosh M. Sinharoy P. Dey S. Bratz I. N. Damron D. S. (2017). TRPA1 ion channel stimulation enhances cardiomyocyte contractile function *via* a CaMKII-dependent pathway. Channels (Austin) 11, 587–603. 10.1080/19336950.2017.1365206 28792844 PMC5786180

[B6] Andrei S. R. Sinharoy P. Bratz I. N. Damron D. S. (2016). TRPA1 is functionally co-expressed with TRPV1 in cardiac muscle: Co-localization at z-discs, costameres and intercalated discs. Channels (Austin) 10, 395–409. 10.1080/19336950.2016.1185579 27144598 PMC4988441

[B7] Aubdool A. A. Kodji X. Abdul-Kader N. Heads R. Fernandes E. S. Bevan S. (2016). TRPA1 activation leads to neurogenic vasodilatation: involvement of reactive oxygen nitrogen species in addition to CGRP and NO. Br. J. Pharmacol. 173, 2419–2433. 10.1111/bph.13519 27189253 PMC4945766

[B8] Bacmeister L. Schwarzl M. Warnke S. Stoffers B. Blankenberg S. Westermann D. (2019). Inflammation and fibrosis in murine models of heart failure. Basic Res. Cardiol. 114, 19. 10.1007/s00395-019-0722-5 30887214

[B9] Bandell M. Story G. M. Hwang S. W. Viswanath V. Eid S. R. Petrus M. J. (2004). Noxious cold ion channel TRPA1 is activated by pungent compounds and bradykinin. Neuron 41, 849–857. 10.1016/S0896-6273(04)00150-3 15046718

[B10] Bautista D. M. Jordt S. E. Nikai T. Tsuruda P. R. Read A. J. Poblete J. (2006). TRPA1 mediates the inflammatory actions of environmental irritants and proalgesic agents. Cell 124, 1269–1282. 10.1016/j.cell.2006.02.023 16564016

[B11] Bers D. M. (2006). Altered cardiac myocyte Ca regulation in heart failure. Physiology (Bethesda) 21, 380–387. 10.1152/physiol.00019.2006 17119150

[B12] Bertin S. Aoki-Nonaka Y. Lee J. de Jong P. R. Kim P. Han T. (2016). The TRPA1 ion channel is expressed in CD4+ T cells and restrains T-cell-mediated colitis through inhibition of TRPV1. Gut. 66, 1584–1596 10.1136/gutjnl-2015-310710 27325418 PMC5173457

[B13] Bodkin J. V. Thakore P. Aubdool A. A. Liang L. Fernandes E. S. Nandi M. (2014). Investigating the potential role of TRPA1 in locomotion and cardiovascular control during hypertension. Pharmacol. Res. Perspect 2, e00052. 10.1002/prp2.52 25505598 PMC4186440

[B14] Brook R. D. Franklin B. Cascio W. Hong Y. Howard G. Lipsett M. (2004). Air pollution and cardiovascular disease: a statement for healthcare professionals from the Expert Panel on Population and Prevention Science of the American Heart Association. Circulation 109, 2655–2671. 10.1161/01.CIR.0000128587.30041.C8 15173049

[B15] Cao D. S. Zhong L. Hsieh T. H. Abooj M. Bishnoi M. Hughes L. (2012). Expression of transient receptor potential ankyrin 1 (TRPA1) and its role in insulin release from rat pancreatic beta cells. PLoS One 7, e38005. 10.1371/journal.pone.0038005 22701540 PMC3365106

[B16] Chen J. Joshi S. K. DiDomenico S. Perner R. J. Mikusa J. P. Gauvin D. M. (2011). Selective blockade of TRPA1 channel attenuates pathological pain without altering noxious cold sensation or body temperature regulation. Pain 152, 1165–1172. 10.1016/j.pain.2011.01.049 21402443

[B17] Conklin D. J. Guo Y. Nystoriak M. A. Jagatheesan G. Obal D. Kilfoil P. J. (2019). TRPA1 channel contributes to myocardial ischemia–reperfusion injury. Am. J. Physiol. Heart Circ. Physiol. 316, H889–H899. 10.1152/ajpheart.00106.2018 30735434 PMC6483018

[B18] Copeland K. W. Boezio A. A. Cheung E. Lee J. Olivieri P. Schenkel L. B. (2014). Development of novel azabenzofuran TRPA1 antagonists as *in vivo* tools. Bioorg. Med. Chem. Lett. 24, 3464–3468. 10.1016/j.bmcl.2014.05.069 24953819

[B19] Corey D. P. Garcia-Anoveros J. Holt J. R. Kwan K. Y. Lin S. Y. Vollrath M. A. (2004). TRPA1 is a candidate for the mechanosensitive transduction channel of vertebrate hair cells. Nature 432, 723–730. 10.1038/nature03066 15483558

[B20] Defalco J. Steiger D. Gustafson A. Emerling D. E. Kelly M. G. Duncton M. A. (2010). Oxime derivatives related to AP18: Agonists and antagonists of the TRPA1 receptor. Bioorg. Med. Chem. Lett. 20, 276–279. 10.1016/j.bmcl.2009.10.113 19945872

[B21] Del C. D. Murphy S. Heiry M. Barrett L. B. Earley T. J. Cook C. A. (2010). TRPA1 contributes to cold hypersensitivity. J. Neurosci. 30, 15165–15174. 10.1523/JNEUROSCI.2580-10.2010 21068322 PMC3021322

[B22] Desai K. Wu L. (2007). Methylglyoxal and advanced glycation endproducts: new therapeutic horizons? Recent Pat. Cardiovasc. Drug Discov. 2, 89–99. 10.2174/157489007780832498 18221107

[B23] Du J. Xie J. Zhang Z. Tsujikawa H. Fusco D. Silverman D. (2010). TRPM7-mediated Ca2+ signals confer fibrogenesis in human atrial fibrillation. Circ. Res. 106, 992–1003. 10.1161/CIRCRESAHA.109.206771 20075334 PMC2907241

[B24] Earley S. (2012). TRPA1 channels in the vasculature. Br. J. Pharmacol. 167, 13–22. 10.1111/j.1476-5381.2012.02018.x 22563804 PMC3448909

[B25] Earley S. Gonzales A. L. Crnich R. (2009). Endothelium-dependent cerebral artery dilation mediated by TRPA1 and Ca2+-Activated K+ channels. Circ. Res. 104, 987–994. 10.1161/CIRCRESAHA.108.189530 19299646 PMC2966339

[B26] Eberhardt M. Dux M. Namer B. Miljkovic J. Cordasic N. Will C. (2014). H2S and NO cooperatively regulate vascular tone by activating a neuroendocrine HNO-TRPA1-CGRP signalling pathway. Nat. Commun. 5, 4381. 10.1038/ncomms5381 25023795 PMC4104458

[B27] Egbuniwe O. Grover S. Duggal A. K. Mavroudis A. Yazdi M. Renton T. (2014). TRPA1 and TRPV4 activation in human odontoblasts stimulates ATP release. J. Dent. Res. 93, 911–917. 10.1177/0022034514544507 25062738 PMC4541108

[B28] Eltzschig H. K. Eckle T. (2011). Ischemia and reperfusion–from mechanism to translation. Nat. Med. 17, 1391–1401. 10.1038/nm.2507 22064429 PMC3886192

[B29] Ferdinandy P. Schulz R. Baxter G. F. (2007). Interaction of cardiovascular risk factors with myocardial ischemia/reperfusion injury, preconditioning, and postconditioning. Pharmacol. Rev. 59, 418–458. 10.1124/pr.107.06002 18048761

[B30] Folkow B. (1982). Physiological aspects of primary hypertension. Physiol. Rev. 62, 347–504. 10.1152/physrev.1982.62.2.347 6461865

[B31] Garcia-Dorado D. Ruiz-Meana M. Inserte J. Rodriguez-Sinovas A. Piper H. M. (2012). Calcium-mediated cell death during myocardial reperfusion. Cardiovasc. Res. 94, 168–180. 10.1093/cvr/cvs116 22499772

[B32] Goldhaber J. I. Philipson K. D. (2013). Cardiac sodium-calcium exchange and efficient excitation–contraction coupling: implications for heart disease. Adv. Exp. Med. Biol. 961, 355–364. 10.1007/978-1-4614-4756-6_30 23224894 PMC3903336

[B33] Graepel R. Fernandes E. S. Aubdool A. A. Andersson D. A. Bevan S. Brain S. D. (2011). 4-oxo-2-nonenal (4-ONE): evidence of transient receptor potential ankyrin 1-dependent and -independent nociceptive and vasoactive responses *in vivo*. J. Pharmacol. Exp. Ther. 337, 117–124. 10.1124/jpet.110.172403 21205926 PMC3063740

[B34] Hazari M. S. Haykal-Coates N. Winsett D. W. Krantz Q. T. King C. Costa D. L. (2011). TRPA1 and sympathetic activation contribute to increased risk of triggered cardiac arrhythmias in hypertensive rats exposed to diesel exhaust. Environ. Health Perspect. 119, 951–957. 10.1289/ehp.1003200 21377951 PMC3223009

[B35] He M. L. Liu W. J. Sun H. Y. Wu W. Liu J. Tse H. F. (2011). Effects of ion channels on proliferation in cultured human cardiac fibroblasts. J. Mol. Cell. Cardiol. 51, 198–206. 10.1016/j.yjmcc.2011.05.008 21620856

[B36] Hooper J. S. Hadley S. H. Morris K. F. Breslin J. W. Dean J. B. Taylor-Clark T. E. (2016). Characterization of cardiovascular reflexes evoked by airway stimulation with allylisothiocyanate, capsaicin, and ATP in Sprague-Dawley rats. J. Appl. Physiol. 120, 580–591. 10.1152/japplphysiol.00944.2015 26718787 PMC4868373

[B37] Jennings R. B. (2013). Historical perspective on the pathology of myocardial ischemia/reperfusion injury. Circ. Res. 113, 428–438. 10.1161/CIRCRESAHA.113.300987 23908330

[B38] Jordt S. E. Bautista D. M. Chuang H. H. McKemy D. D. Zygmunt P. M. Hogestatt E. D. (2004). Mustard oils and cannabinoids excite sensory nerve fibres through the TRP channel ANKTM1. Nature 427, 260–265. 10.1038/nature02282 14712238

[B39] Katz A. M. Rolett E. L. (2016). Heart failure: when form fails to follow function. Eur. Heart J. 37, 449–454. 10.1093/eurheartj/ehv548 26497163

[B40] Khan R. Spagnoli V. Tardif J. C. L’Allier P. L. (2015). Novel anti-inflammatory therapies for the treatment of atherosclerosis. Atherosclerosis 240, 497–509. 10.1016/j.atherosclerosis.2015.04.783 25917947

[B41] Khera A. V. Cuchel M. de la Llera-Moya M. Rodrigues A. Burke M. F. Jafri K. (2011). Cholesterol efflux capacity, high-density lipoprotein function, and atherosclerosis. N. Engl. J. Med. 364, 127–135. 10.1056/NEJMoa1001689 21226578 PMC3030449

[B42] Kojima R. Nozawa K. Doihara H. Keto Y. Kaku H. Yokoyama T. (2014). Effects of novel TRPA1 receptor agonist ASP7663 in models of drug-induced constipation and visceral pain. Eur. J. Pharmacol. 723, 288–293. 10.1016/j.ejphar.2013.11.020 24291101

[B43] Kokel D. Cheung C. Y. Mills R. Coutinho-Budd J. Huang L. Setola V. (2013). Photochemical activation of TRPA1 channels in neurons and animals. Nat. Chem. Biol. 9, 257–263. 10.1038/nchembio.1183 23396078 PMC3604056

[B44] Kunkler P. E. Ballard C. J. Oxford G. S. Hurley J. H. (2011). TRPA1 receptors mediate environmental irritant-induced meningeal vasodilatation. Pain 152, 38–44. 10.1016/j.pain.2010.08.021 21075522 PMC3012007

[B45] Kurhanewicz N. Ledbetter A. Farraj A. Hazari M. (2018). TRPA1 mediates the cardiac effects of acrolein through parasympathetic dominance but also sympathetic modulation in mice. Toxicol. Appl. Pharmacol. 347, 104–114. 10.1016/j.taap.2018.03.027 29627347 PMC6220342

[B46] Kurhanewicz N. Intosh-Kastrinsky R. Tong H. Ledbetter A. Walsh L. Farraj A. (2016). TRPA1 mediates changes in heart rate variability and cardiac mechanical function in mice exposed to acrolein. Toxicol. Appl. Pharmacol. 324, 51–60 10.1016/j.taap.2016.10.008 PMC5391294 27746315

[B47] Langrish J. P. Watts S. J. Hunter A. J. Shah A. S. Bosson J. A. Unosson J. (2014). Controlled exposures to air pollutants and risk of cardiac arrhythmia. Environ. Health Perspect. 122, 747–753. 10.1289/ehp.1307337 24667535 PMC4080532

[B48] Lau D. H. Nattel S. Kalman J. M. Sanders P. (2017). Modifiable Risk Factors and Atrial Fibrillation. Circulation 136, 583–596. 10.1161/CIRCULATIONAHA.116.023163 28784826

[B49] Leffler A. Lattrell A. Kronewald S. Niedermirtl F. Nau C. (2011). Activation of TRPA1 by membrane permeable local anesthetics. Mol. Pain 7, 62. 10.1186/1744-8069-7-62 21861907 PMC3179737

[B50] Libby P. (2002). Inflammation in atherosclerosis. Nature 420, 868–874. 10.1038/nature01323 12490960

[B51] Lu Y. Piplani H. McAllister S. L. Hurt C. M. Gross E. R. (2016). Transient Receptor Potential Ankyrin 1 Activation within the Cardiac Myocyte Limits Ischemia–reperfusion Injury in Rodents. Anesthesiology 125, 1171–1180. 10.1097/ALN.0000000000001377 27748654 PMC5110384

[B52] Macpherson L. J. Geierstanger B. H. Viswanath V. Bandell M. Eid S. R. Hwang S. (2005). The pungency of garlic: activation of TRPA1 and TRPV1 in response to allicin. Curr. Biol. 15, 929–934. 10.1016/j.cub.2005.04.018 15916949

[B53] Mancia G. Grassi G. (2014). The autonomic nervous system and hypertension. Circ. Res. 114, 1804–1814. 10.1161/CIRCRESAHA.114.302524 24855203

[B54] Marks A. R. (2013). Calcium cycling proteins and heart failure: mechanisms and therapeutics. J. Clin. Invest. 123, 46–52. 10.1172/JCI62834 23281409 PMC3533269

[B55] McNamara C. R. Mandel-Brehm J. Bautista D. M. Siemens J. Deranian K. L. Zhao M. (2007). TRPA1 mediates formalin-induced pain. Proc. Natl. Acad. Sci. U. S. A. 104, 13525–13530. 10.1073/pnas.0705924104 17686976 PMC1941642

[B56] Middlekauff H. R. Park J. Moheimani R. S. (2014). Adverse effects of cigarette and noncigarette smoke exposure on the autonomic nervous system: mechanisms and implications for cardiovascular risk. J. Am. Coll. Cardiol. 64, 1740–1750. 10.1016/j.jacc.2014.06.1201 25323263

[B57] Molkentin J. D. Lu J. R. Antos C. L. Markham B. Richardson J. Robbins J. (1998). A calcineurin-dependent transcriptional pathway for cardiac hypertrophy. Cell 93, 215–228. 10.1016/S0092-8674(00)81573-1 9568714 PMC4459646

[B58] Momiyama Y. Adachi H. Fairweather D. Ishizaka N. Saita E. (2014). Inflammation, Atherosclerosis and Coronary Artery Disease. Clin. Med. Insights Cardiol. 8, 67–70. 10.4137/CMC.S39423 PMC5003124 27594791

[B59] Moore K. J. Sheedy F. J. Fisher E. A. (2013). Macrophages in atherosclerosis: a dynamic balance. Nat. Rev. Immunol. 13, 709–721. 10.1038/nri3520 23995626 PMC4357520

[B60] Moore-Morris T. Guimaraes-Camboa N. Yutzey K. E. Puceat M. Evans S. M. (2015). Cardiac fibroblasts: from development to heart failure. J. Mol. Med. (Berl.) 93, 823–830. 10.1007/s00109-015-1314-y 26169532 PMC4512919

[B61] Mukhopadhyay I. Gomes P. Aranake S. Shetty M. Karnik P. Damle M. (2011). Expression of functional TRPA1 receptor on human lung fibroblast and epithelial cells. J. Recept. Signal Transduct. Res. 31, 350–358. 10.3109/10799893.2011.602413 21848366

[B62] Murphy E. Steenbergen C. (2008). Mechanisms underlying acute protection from cardiac ischemia–reperfusion injury. Physiol. Rev. 88, 581–609. 10.1152/physrev.00024.2007 18391174 PMC3199571

[B63] Nagata K. Duggan A. Kumar G. Garcia-Anoveros J. (2005). Nociceptor and hair cell transducer properties of TRPA1, a channel for pain and hearing. J. Neurosci. 25, 4052–4061. 10.1523/JNEUROSCI.0013-05.2005 15843607 PMC6724946

[B64] Nassini R. Pedretti P. Moretto N. Fusi C. Carnini C. Facchinetti F. (2012). Transient receptor potential ankyrin 1 channel localized to non-neuronal airway cells promotes non-neurogenic inflammation. PLoS One 7, e42454. 10.1371/journal.pone.0042454 22905134 PMC3419223

[B65] Nilius B. Appendino G. Owsianik G. (2012). The transient receptor potential channel TRPA1: from gene to pathophysiology. Pflugers Arch. 464, 425–458. 10.1007/s00424-012-1158-z 23001121

[B66] Nilius B. Owsianik G. (2011). The transient receptor potential family of ion channels. Genome Biol. 12, 218. 10.1186/gb-2011-12-3-218 21401968 PMC3129667

[B67] Nilius B. Szallasi A. (2014). Transient receptor potential channels as drug targets: from the science of basic research to the art of medicine. Pharmacol. Rev. 66, 676–814. 10.1124/pr.113.008268 24951385

[B68] Nozawa K. Kawabata-Shoda E. Doihara H. Kojima R. Okada H. Mochizuki S. (2009). TRPA1 regulates gastrointestinal motility through serotonin release from enterochromaffin cells. Proc. Natl. Acad. Sci. U. S. A. 106, 3408–3413. 10.1073/pnas.0805323106 19211797 PMC2651261

[B69] Numaga-Tomita T. Oda S. Shimauchi T. Nishimura A. Mangmool S. Nishida M. (2017). TRPC3 Channels in Cardiac Fibrosis. Front. Cardiovasc. Med. 4, 56. 10.3389/fcvm.2017.00056 28936433 PMC5594069

[B70] Oguri G. Nakajima T. Yamamoto Y. Takano N. Tanaka T. Kikuchi H. (2014). Effects of methylglyoxal on human cardiac fibroblast: roles of transient receptor potential ankyrin 1 (TRPA1) channels. Am. J. Physiol. Heart Circ. Physiol. 307, H1339–H1352. 10.1152/ajpheart.01021.2013 25172898

[B71] Okada Y. Shirai K. Reinach P. S. Kitano-Izutani A. Miyajima M. Flanders K. C. (2014). TRPA1 is required for TGF-beta signaling and its loss blocks inflammatory fibrosis in mouse corneal stroma. Lab. Invest. 94, 1030–1041. 10.1038/labinvest.2014.85 25068659 PMC5919187

[B72] Oparil S. Schmieder R. E. (2015). New approaches in the treatment of hypertension. Circ. Res. 116, 1074–1095. 10.1161/CIRCRESAHA.116.303603 25767291

[B73] Perez C. M. Hazari M. S. Farraj A. K. (2015). Role of autonomic reflex arcs in cardiovascular responses to air pollution exposure. Cardiovasc. Toxicol. 15, 69–78. 10.1007/s12012-014-9272-0 25123706 PMC4766835

[B74] Petrus M. Peier A. M. Bandell M. Hwang S. W. Huynh T. Olney N. (2007). A role of TRPA1 in mechanical hyperalgesia is revealed by pharmacological inhibition. Mol. Pain 3, 40. 10.1186/1744-8069-3-40 18086313 PMC2222610

[B75] Pires P. W. Earley S. (2017). Redox regulation of transient receptor potential channels in the endothelium. Microcirculation 24. 10.1111/micc.12329 PMC5404942 27809396

[B76] Pozsgai G. Bodkin J. V. Graepel R. Bevan S. Andersson D. A. Brain S. D. (2010). Evidence for the pathophysiological relevance of TRPA1 receptors in the cardiovascular system *in vivo*. Cardiovasc. Res. 87, 760–768. 10.1093/cvr/cvq118 20442136

[B77] Qian X. Francis M. Solodushko V. Earley S. Taylor M. S. (2013). Recruitment of dynamic endothelial Ca2+ signals by the TRPA1 channel activator AITC in rat cerebral arteries. Microcirculation 20, 138–148. 10.1111/micc.12004 22928941 PMC3524345

[B78] Raedschelders K. Ansley D. M. Chen D. D. (2012). The cellular and molecular origin of reactive oxygen species generation during myocardial ischemia and reperfusion. Pharmacol. Ther. 133, 230–255. 10.1016/j.pharmthera.2011.11.004 22138603

[B79] Roe A. T. Frisk M. Louch W. E. (2015). Targeting cardiomyocyte Ca2+ homeostasis in heart failure. Curr. Pharm. Des. 21, 431–448. 10.2174/138161282104141204124129 25483944 PMC4475738

[B80] Rooney L. Vidal A. D’Souza A. M. Devereux N. Masick B. Boissel V. (2014). Discovery, optimization, and biological evaluation of 5-(2-(trifluoromethyl)phenyl) indazoles as a novel class of transient receptor potential A1 (TRPA1) antagonists. J. Med. Chem. 57, 5129–5140. 10.1021/jm401986p 24884675

[B81] Schirone L. Forte M. Palmerio S. Yee D. Nocella C. Angelini F. (2017). A Review of the Molecular Mechanisms Underlying the Development and Progression of Cardiac Remodeling. Oxid. Med. Cell. Longev. 2017, 3920195. 10.1155/2017/3920195 28751931 PMC5511646

[B82] Shang S. Zhu F. Liu B. Chai Z. Wu Q. Hu M. (2016). Intracellular TRPA1 mediates Ca2+ release from lysosomes in dorsal root ganglion neurons. J. Cell Biol. 215, 369–381. 10.1083/jcb.201603081 27799370 PMC5100290

[B83] Solanki A. Bhatt L. K. Johnston T. P. (2018). Evolving targets for the treatment of atherosclerosis. Pharmacol. Ther. 187, 1–12. 10.1016/j.pharmthera.2018.02.002 29414673

[B84] Stevens J. F. Maier C. S. (2008). Acrolein: sources, metabolism, and biomolecular interactions relevant to human health and disease. Mol. Nutr. Food Res. 52, 7–25. 10.1002/mnfr.200700412 18203133 PMC2423340

[B85] Story G. M. Peier A. M. Reeve A. J. Eid S. R. Mosbacher J. Hricik T. R. (2003). ANKTM1, a TRP-like channel expressed in nociceptive neurons, is activated by cold temperatures. Cell 112, 819–829. 10.1016/S0092-8674(03)00158-2 12654248

[B86] Subramanian U. Nagarajan D. (2017). All-Trans Retinoic Acid supplementation prevents cardiac fibrosis and cytokines induced by Methylglyoxal. Glycoconj. J. 34, 255–265. 10.1007/s10719-016-9760-5 28091942

[B87] Takahashi N. Kuwaki T. Kiyonaka S. Numata T. Kozai D. Mizuno Y. (2011). TRPA1 underlies a sensing mechanism for O2. Nat. Chem. Biol. 7, 701–711. 10.1038/nchembio.640 21873995

[B88] Talavera K. Gees M. Karashima Y. Meseguer V. M. Vanoirbeek J. A. Damann N. (2009). Nicotine activates the chemosensory cation channel TRPA1. Nat. Neurosci. 12, 1293–1299. 10.1038/nn.2379 19749751

[B89] Taler S. J. (2018). Initial Treatment of Hypertension. N. Engl. J. Med. 378, 636–644. 10.1056/NEJMcp1613481 29443671

[B90] Taylor-Clark T. E. McAlexander M. A. Nassenstein C. Sheardown S. A. Wilson S. Thornton J. (2008). Relative contributions of TRPA1 and TRPV1 channels in the activation of vagal bronchopulmonary C-fibres by the endogenous autacoid 4-oxononenal. J. Physiol. 586, 3447–3459. 10.1113/jphysiol.2008.153585 18499726 PMC2538817

[B91] Thodeti C. K. Paruchuri S. Meszaros J. G. (2013). A TRP to cardiac fibroblast differentiation. Channels (Austin) 7, 211–214. 10.4161/chan.24328 23511028 PMC3710348

[B92] Thompson L. C. Walsh L. Martin B. L. McGee J. Wood C. Kovalcik K. (2019). Ambient Particulate Matter and Acrolein Co-Exposure Increases Myocardial Dyssynchrony in Mice *via* TRPA1. Toxicol. Sci. 167, 559–572. 10.1093/toxsci/kfy262 30351402

[B93] Trevisani M. Siemens J. Materazzi S. Bautista D. M. Nassini R. Campi B. (2007). 4-Hydroxynonenal, an endogenous aldehyde, causes pain and neurogenic inflammation through activation of the irritant receptor TRPA1. Proc. Natl. Acad. Sci. U. S. A. 104, 13519–13524. 10.1073/pnas.0705923104 17684094 PMC1948902

[B94] Venkatachalam K. Montell C. (2007). TRP channels. Annu. Rev. Biochem. 76, 387–417. 10.1146/annurev.biochem.75.103004.142819 17579562 PMC4196875

[B95] Viana F. (2016). TRPA1 channels: molecular sentinels of cellular stress and tissue damage. J. Physiol. 594, 4151–4169. 10.1113/JP270935 27079970 PMC4967735

[B96] Wang Y. Y. Chang R. B. Waters H. N. McKemy D. D. Liman E. R. (2008). The nociceptor ion channel TRPA1 is potentiated and inactivated by permeating calcium ions. J. Biol. Chem. 283, 32691–32703. 10.1074/jbc.M803568200 18775987 PMC2583289

[B97] Wang Z. Wang M. Liu J. Ye J. Jiang H. Xu Y. (2018a). Inhibition of TRPA1 Attenuates Doxorubicin-Induced Acute Cardiotoxicity by Suppressing Oxidative Stress, the Inflammatory Response, and Endoplasmic Reticulum Stress. Oxid. Med. Cell. Longev. 2018, 5179468. 10.1155/2018/5179468 29682158 PMC5850896

[B98] Wang Z. Xu Y. Wang M. Ye J. Liu J. Jiang H. (2018b). TRPA1 inhibition ameliorates pressure overload-induced cardiac hypertrophy and fibrosis in mice. EBioMedicine. 36, 54–62. 10.1016/j.ebiom.2018.08.022 30297144 PMC6197736

[B99] Wei H. Hamalainen M. M. Saarnilehto M. Koivisto A. Pertovaara A. (2009). Attenuation of mechanical hypersensitivity by an antagonist of the TRPA1 ion channel in diabetic animals. Anesthesiology 111, 147–154. 10.1097/ALN.0b013e3181a1642b 19512877

[B100] Woll K. A. Skinner K. A. Gianti E. Bhanu N. V. Garcia B. A. Carnevale V. (2017). Sites Contributing to TRPA1 Activation by the Anesthetic Propofol Identified by Photoaffinity Labeling. Biophys. J. 113, 2168–2172. 10.1016/j.bpj.2017.08.040 28935134 PMC5700380

[B101] Yamamoto S. Shimizu S. (2016). Significance of TRP channels in oxidative stress. Eur. J. Pharmacol. 793, 109–111. 10.1016/j.ejphar.2016.11.007 27838397

[B102] Ye J. Ji Q. Liu J. Liu L. Huang Y. Shi Y. (2017a). Interleukin 22 Promotes Blood Pressure Elevation and Endothelial Dysfunction in Angiotensin II-Treated Mice. J. Am. Heart Assoc. 6. 10.1161/JAHA.117.005875 PMC5721831 28974499

[B103] Ye J. Wang M. Xu Y. Liu J. Jiang H. Wang Z. (2017b). Sestrins increase in patients with coronary artery disease and associate with the severity of coronary stenosis. Clin. Chim. Acta 472, 51–57. 10.1016/j.cca.2017.07.020 28732653

[B104] Ye J. Wang Z. Ye D. Wang Y. Wang M. Ji Q. (2019). Increased Interleukin-11 Levels Are Correlated with Cardiac Events in Patients with Chronic Heart Failure. Mediators Inflamm. 2019, 1575410. 10.1155/2019/1575410 30728748 PMC6341241

[B105] Yue Z. Zhang Y. Xie J. Jiang J. Yue L. (2013). Transient receptor potential (TRP) channels and cardiac fibrosis. Curr. Top. Med. Chem. 13, 270–282. 10.2174/1568026611313030005 23432060 PMC3874073

[B106] Zhang N. Wei W. Y. Li L. L. Hu C. Tang Q. Z. (2018). Therapeutic Potential of Polyphenols in Cardiac Fibrosis. Front. Pharmacol. 9, 122. 10.3389/fphar.2018.00122 29497382 PMC5818417

[B107] Zhao J. F. Shyue S. K. Kou Y. R. Lu T. M. Lee T. S. (2016). Transient Receptor Potential Ankyrin 1 Channel Involved in Atherosclerosis and Macrophage-Foam Cell Formation. Int. J. Biol. Sci. 12, 812–823. 10.7150/ijbs.15229 27313495 PMC4910600

[B108] Zhou T. Chuang C. C. Zuo L. (2015). Molecular Characterization of Reactive Oxygen Species in Myocardial Ischemia-Reperfusion Injury. Biomed Res. Int. 2015, 864946. 10.1155/2015/864946 26509170 PMC4609796

[B109] Zurborg S. Yurgionas B. Jira J. A. Caspani O. Heppenstall P. A. (2007). Direct activation of the ion channel TRPA1 by Ca2+. Nat. Neurosci. 10, 277–279. 10.1038/nn1843 17259981

[B110] Zygmunt P. M. Hogestatt E. D. (2014). TRPA1. Handb. Exp. Pharmacol. 222, 583–630. 10.1007/978-3-642-54215-2_23z 24756722

